# A Blunted Sympathetic Function and an Enhanced Nitrergic Activity Contribute to Reduce Mesenteric Resistance in Hyperthyroidism

**DOI:** 10.3390/ijms22020570

**Published:** 2021-01-08

**Authors:** Laia Cros-Brunsó, Laura Camacho-Rodríguez, Ángel Martínez-González, Pablo Llévenes, Mercedes Salaices, Ana Belen García-Redondo, Javier Blanco-Rivero

**Affiliations:** 1 Department of Physiology, School of Medicine, Universidad Autónoma de Madrid, Calle de Arzobispo Morcillo 4, 28029 Madrid, Spain; croslaia@gmail.com (L.C.-B.); lauri166@gmail.com (L.C.-R.); mgangel9797@gmail.com (Á.M.-G.); pablollevenes@gmail.com (P.L.); 2 Department of Pharmacology and Therapeutics, School of Medicine, Universidad Autónoma de Madrid, Calle de Arzobispo Morcillo 4, 28029 Madrid, Spain; mercedes.salaices@uam.es; 3 Research Institute University Hospital la Paz (IdIPaz), Calle de Pedro Rico 6, 28029 Madrid, Spain; 4 Center for Biomedical Research Network in Cardiovascular Diseases (CIBERCV), Calle de Melchor Fernández Almagro 3, 28029 Madrid, Spain

**Keywords:** hyperthyroidism, rat mesenteric artery, perivascular innervation, noradrenaline, neuronal nitric oxide, protein kinase C, PI3K/AKT pathway

## Abstract

We aimed to determine whether an experimental model of hyperthyroidism could alter the function of sympathetic and nitrergic components of mesenteric innervation. For this purpose, male Wistar rats were divided into (1) control rats (CT) and (2) rats infused with L-Thyroxine (HT). Body weight gain and adipose tissue accumulation were lower in HT rats, while systolic blood pressure and citrate synthase activity in the soleus muscle were increased by HT. In segments from the superior mesenteric artery, the application of an electrical field stimulation (EFS) induced a vasoconstrictor response, which was lower in arteries from HT animals. The alpha-adrenoceptor antagonist phentolamine diminished EFS-induced vasoconstriction to a lower extent in HT arteries, while the purinergic receptor antagonist suramin reduced contractile response to EFS only in segments from CT. In line with this, noradrenaline release, tyrosine hydroxylase expression and activation and dopamine β hydroxylase expression were diminished in HT. The unspecific nitric oxide synthase (NOS) inhibitor L-NAME increased EFS-induced vasoconstriction more markedly in segments from HT rats. NO release was enhanced in HT, probably due to an enhancement in neuronal NOS activity, in which a hyperactivation of both PKC and PI3K-AKT signaling pathways might play a relevant role. In conclusion, perivascular mesenteric innervation might contribute to reduce the vascular resistance observed in hyperthyroidism.

## 1. Introduction

Thyroid hormones mediate a wide range of important physiological effects, regulating different growth and metabolic pathways that affect almost every tissue and organ [1,2]. Many known factors and pathologies, either inherent to the thyroid gland or to a non-thyroidal origin, can lead to hyperthyroidism [3], which appears in 0.8% of the population in Europe and 1.3% of the population in the USA [4]. When this pathology appears, the excessive production of thyroid hormones influences the different organs and tissues, including complex actions in the cardiovascular system [5]. Hence, hyperthyroidism causes a hyperdynamic cardiac state, characterized by sinus tachycardia and increased left ventricular contraction and relaxation, leading to an enhanced systolic blood pressure [6]. Conversely, hyperthyroidism is associated to reduced systemic vascular resistance through either a direct vascular smooth muscle cell (VSMC) relaxation [7] or due to an enhanced nitric oxide (NO), adrenomedullin and adenosine production in both endothelia and VSMCs [8,9,10]. In addition, hyperthyroidism can alter the release and/or function of other vasoactive factors, such as endothelin-1 or angiotensin-II, thereby modulating vascular resistance [11,12]. Consequently, hyperthyroidism can be considered a model of hypertension with a cardiac origin [13].

Hyperthyroidism can have an autoimmune origin, mainly through a potentiation of Th1 cell activity [14,15]. In line with this, different types of immune cells coexist in the peritoneal cavity and can migrate to the splanchnic vasculature in response to different inflammatory stimuli, consequently modulating the mesenteric vascular resistance through the release of different chemokines [16]. Several studies from our group have linked an increase in different interleukins, enhanced in hyperthyroidism, with a structural remodeling in mesenteric vasculature, thereby altering systemic vascular resistance [15,17,18].

Another factor implicated in the regulation of vascular resistance is perivascular innervation, which plays a major role in certain vessels such as the mesenteric vascular bed, where blood flow is approximately 20–30% of the total cardiac output. Inadequate mesenteric blood flow and tissue perfusion can induce relevant hemodynamic changes [19,20]. The superior mesenteric artery (SMA) has rich and functional sympathetic and nitrergic nerve endings [21,22], which release noradrenaline (NA) and ATP, and NO, respectively. The implication of each of these innervations depends on the synthesis, release, response and metabolism of the different neurotransmitters, which have been reported to change in different pathophysiological situations. In this context, we reported that pathophysiological alterations in hormone status (pregnancy, breast feeding, diabetes, glucocorticoids, thyroid hormone exposure) can modify the role of the different perivascular innervation components in this artery [23,24,25,26,27].

It is widely known that most of the symptoms related to hyperthyroidism (tachycardia, sweating) have been associated to an adrenergic hyperactivity, which is related to the enhanced circulating catecholamines that have been reported in hyperthyroidic patients [28,29]. Regarding NO, enhanced circulating NO derivatives NOx have been reported in patients with hyperthyroidism [30]. Additionally, thyroid hormones can either enhance or decrease neuronal nitric oxide synthase (nNOS) expression and activity in different tissues, including the perivascular nervous system and VSMC, through the hyperactivation of PI3K-AKT, PKA and/or PKC signaling pathways [1,8,27,31,32].

Although it is well known that hyperthyroidism leads to decreased vascular resistance, the mechanisms involved in this event are not fully understood. Given the relevant role that perivascular innervation plays in regulating the mesenteric vascular tone, we hypothesize that this innervation might take part in the diminished vascular resistance observed in hyperthyroidism. Hence, the aim of this study was to analyze possible alterations in the sympathetic and nitrergic components on mesenteric innervation in an experimental model of hyperthyroidism.

## 2. Results

### 2.1. Animal Evolution

An increase in systolic blood pressure was found in L-Thyroxine-treated rats compared to control (CT) animals, without an alteration in heart size. In addition, despite the fact we found no differences in soleus weight, an increase in the metabolic activity marker citrate synthase was found in the soleus muscle from rats treated with L-Thyroxine. According to the increased metabolic rate and reduced body weight gain observed in L-Thyroxine-treated animals, a reduction in adipose tissue was found (Table 1). Altogether, these results show that treatment with L-Thyroxine for two weeks is a valid model for the development of hyperthyroidism (HT).

### 2.2. Effect of Hyperthyroidism in Electrical Field Stimulation (EFS)-Induced Vasoconstriction

**Figure 1:**
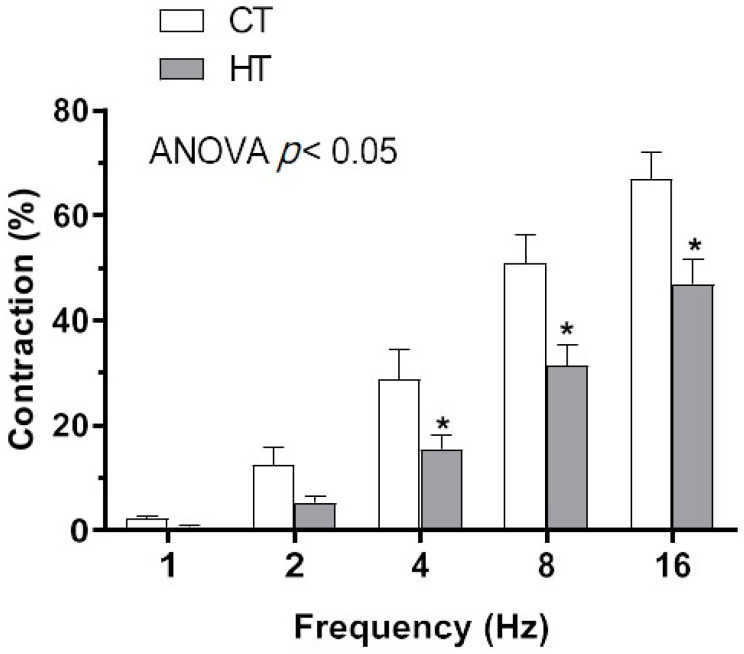
Electrical field stimulation (EFS)-induced vasoconstriction in mesenteric segments from control (CT) and hyperthyroidic (HT) Wistar rats. Results (mean ± S.E.M.) are expressed as a percentage of the previous tone elicited by KCl. *n* = 5–6 segments from different rats in each experimental group. * *p* < 0.05 CT vs. HT at each frequency (Bonferroni post hoc test).

Perivascular mesenteric innervation plays a relevant role in generating and maintaining mesenteric vascular resistance [21,22]. We have previously reported that the endothelium-derived vasoactive factors angiotensin II or endothelin, whose function can be altered by hyperthyroidism, can modify the perivascular innervation function [11,12,33,34]. Hence, we used endothelium-denuded segments from the rat mesenteric artery to determine whether HT might alter the function of perivascular mesenteric innervation. When applying an EFS, we observed a frequency-dependent contraction in endothelium-denuded arteries from both experimental groups. This constriction was lower in segments from HT rats (Figure 1).

The fact that preincubation with the nerve impulse propagation blocker TTX abolished EFS-induced contraction in segments from both experimental groups (Table 2) confirmed the neural origin of this EFS-induced vasoconstriction.

### 2.3. Effect of Hyperthyroidism in the Sympathetic Neurotransduction in Mesenteric Vascular Innervation

**Figure 2:**
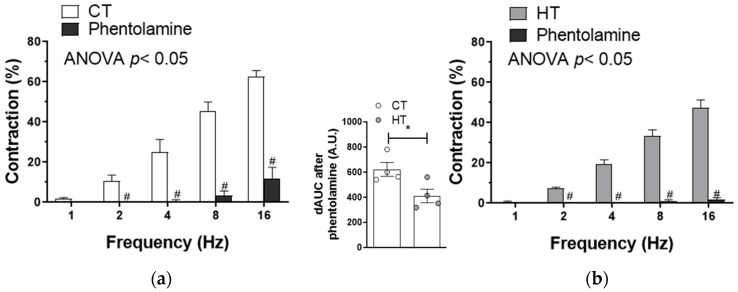
Effect of alpha-adrenergic antagonist phentolamine (1 µmol/L) on EFS-induced vasoconstriction in mesenteric segments from control (CT; (**a**)) and rats with hyperthyroidism (HT, (**b**)). Results (mean ± S.E.M.) are expressed as a percentage of previous tone induced by KCl. # *p <* 0.05 conditions without drug vs. phentolamine at each frequency (Bonferroni post hoc test). *n* = 4 segments from different rats in each experimental group. Insert graph shows differences in the area under the curve (dAUC) in presence/absence of phentolamine. * *p <* 0.05 CT vs. HT (Student’s *t*-test).

Sympathetic innervation is responsible for most of the typical symptoms related to hyperthyroidism, such as tachycardia and sweating [28]. The SMA has rich and functional sympathetic innervation. When analyzing the role of the adrenergic component of sympathetic innervation, we found that preincubation with the α-adrenoceptor antagonist phentolamine (1 μmol/L) decreased the vasoconstrictor response induced by EFS in endothelium-denuded segments from both experimental groups (Figure 2a,b). Although phentolamine abolished EFS-induced vasoconstriction in HT, the analysis of the differences in the area under the curve (dAUC) analysis showed that this decrease was lower in mesenteric segments from HT animals.

**Figure 3:**
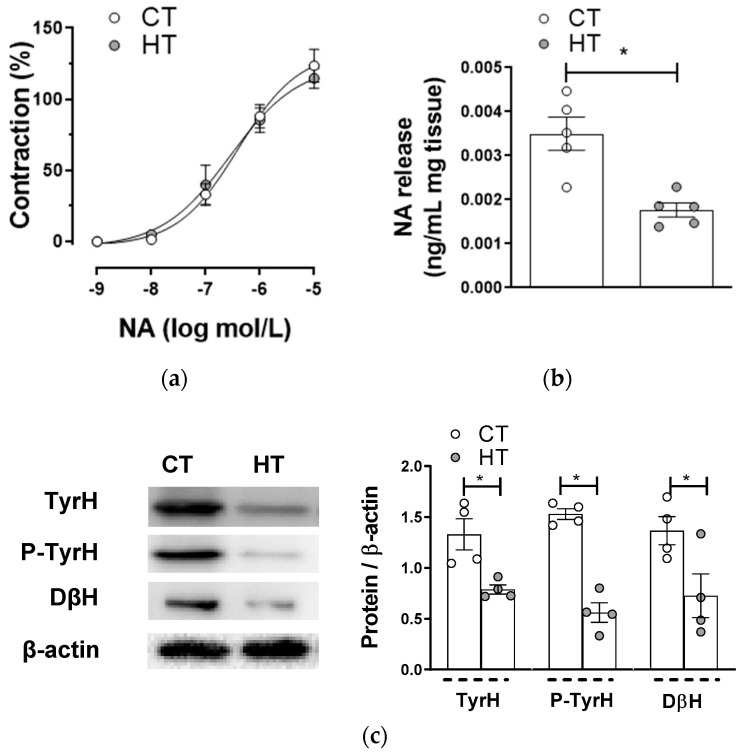
(**a**) Vasoconstrictor response to exogenous noradrenaline (NA) in mesenteric arteries from control (CT) and hyperthyroidic (HT) rats. Results (mean ± S.E.M.) are expressed as a percentage of the previous tone elicited by KCl. *n* = 4-6 segments from different animals in each experimental group. (**b**) EFS-induced NA release in mesenteric segments from CT and HT. Results (mean ± S.E.M.) are expressed as ng NA/mL mg tissue. *n* = 5 animals per group. * *p <* 0.05 CT vs. HT (Student’s t-test). (**c**) Effect of hyperthyroidism on tyrosine hydroxylase (TH) expression and activation (P-TyrH), and in dopamine β hydroxylase (DβH) expression. The blots are representative of four separate segments from each group. Right panel shows a relation between each protein expression and β-actin. Results (mean ± S.E.M.) are expressed as a ratio of the signal obtained for each protein and the signal obtained for β-actin. * *p <* 0.05 CT vs. HT (Student’s *t*-test).

Alterations in either VSMC sensitivity to NA or the release of NA can be responsible for the lower EFS-induced vasoconstriction observed in HT. NA-induced vasoconstriction was similar in both experimental groups (Figure 3a), while EFS-induced NA release was lower in mesenteric segments from HT animals compared with segments from CT animals (Figure 3b). In addition, a decrease in tyrosine hydroxylase (TyrH) and dopamine β hydroxylase (DβH) expressions was observed in segments from HT rats. The activation of TyrH, through phosphorylation in the Ser40 residue (P-TyrH), was also diminished in arteries from HT animals (Figure 3c). These results indicate that the diminished adrenergic component in the vasoconstrictor response induced by EFS, observed in mesenteric rings from HT rats, is due to a blunted NA synthesis and release.

**Figure 4:**
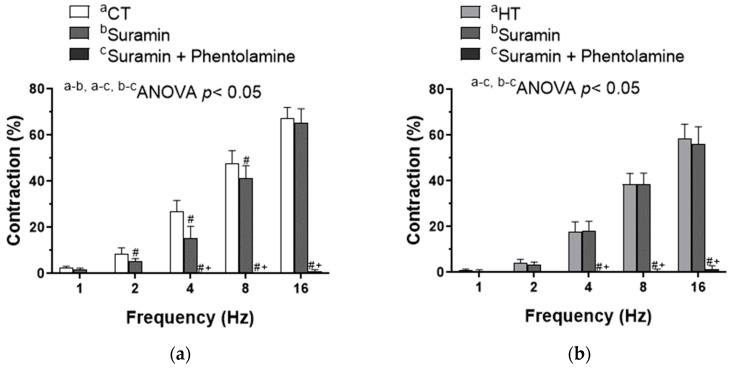
Effect of preincubation with 0.1 mmol/L suramin (antagonist of P2 purinergic receptors) or a combination of phentolamine + suramin on the vasoconstriction response induced by EFS in mesenteric segments from control (CT, (**a**)) and rats with hyperthyroidism (HT, (**b**)). Results (mean ± S.E.M.) are expressed as a percentage of the initial contraction elicited by KCl. *n* = 5–6 animals in each group. # *p <* 0.05 conditions without drug vs. conditions with suramin at each frequency (Bonferroni post hoc test). + *p <* 0.05 conditions without drug vs. conditions with phentolamine + suramin at each frequency (Bonferroni post hoc test).

Sympathetic neurotransmission involves simultaneous NA and ATP release, both acting on VSMC. Preincubation with 0.1 mmol/L suramin, a non-specific P2 purinergic receptor antagonist, decreased EFS-induced contraction only in CT segments. Moreover, preincubation with phentolamine plus suramin abolished this EFS-induced contraction in CT segments from both groups (Figure 4a,b). To sum up, the abolishment of purinergic co-transmission observed in HT might also participate in the diminished sympathetic function in superior mesenteric arteries from these animals.

### 2.4. Participation of the Nitrergic Component in Vascular Responses to EFS

**Figure 5:**
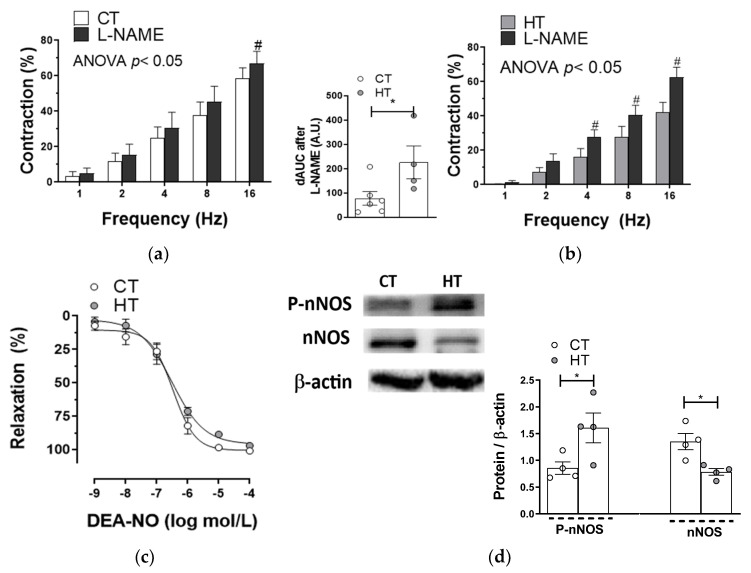
Effect of preincubation with the unspecific nitric oxide synthase (NOS) inhibitor L-NAME on EFS-induced vasoconstriction in mesenteric arteries from control (CT, (**a**)) and rats with hyperthyroidism (HT, (**b**)). Results (mean ± S.E.M.) are expressed as a percentage of previous tone induced by KCl. # *p <* 0.05 conditions without drug vs. L-NAME at each frequency (Bonferroni post hoc test). *n* = 4–6 segments from different animals in each experimental group. Insert graph represents the differences in the area under the curve (dAUC) in presence/absence of L-NAME. * *p <* 0.05 CT vs. HT (Student’s t-test). (**c**) Vasodilator response to NO donor DEA-NO in mesenteric segments from CT and HT rats. Results (mean ± S.E.M.) are expressed as a percentage of the previous tone elicited by noradrenaline. *n* = 5 segments from different animals in each experimental group. (**d**) Expression and phosphorylation on Ser 1417 of nNOS in mesenteric rings from CT and HT rats (4 isolated segments from each group). Lower panels show densitometry analysis for the expression of each protein. Results (mean + S.E.M.) are expressed as the relation between the signal obtained for the analyzed protein and the signal obtained for β-actin. * *p <* 0.05 CT vs. HT (Student’s *t*-test).

The nitrergic component in perivascular mesenteric innervation has a vasodilator role that helps to decrease the contractile response to EFS. Among the mechanisms by which thyroid hormones diminish vascular resistance, an increased production of NO has been described [8,27]. Preincubation with the non-specific NOS inhibitor Nω-Nitro-L-arginine methyl ester (L-NAME, 0.1 mmol/L) significantly increased the EFS contractile response in endothelium-denuded segments from both experimental groups (Figure 5a,b). The analysis of dAUC showed that this increase was greater in segments from HT animals, suggesting a greater role of nitrergic innervation in this pathology. This effect was not due to a greater VSMC sensitivity to NO, since the vasodilator response to exogenous NO donor DEA-NO was similar in arteries from both experimental groups (Figure 5c).

**Figure 6:**
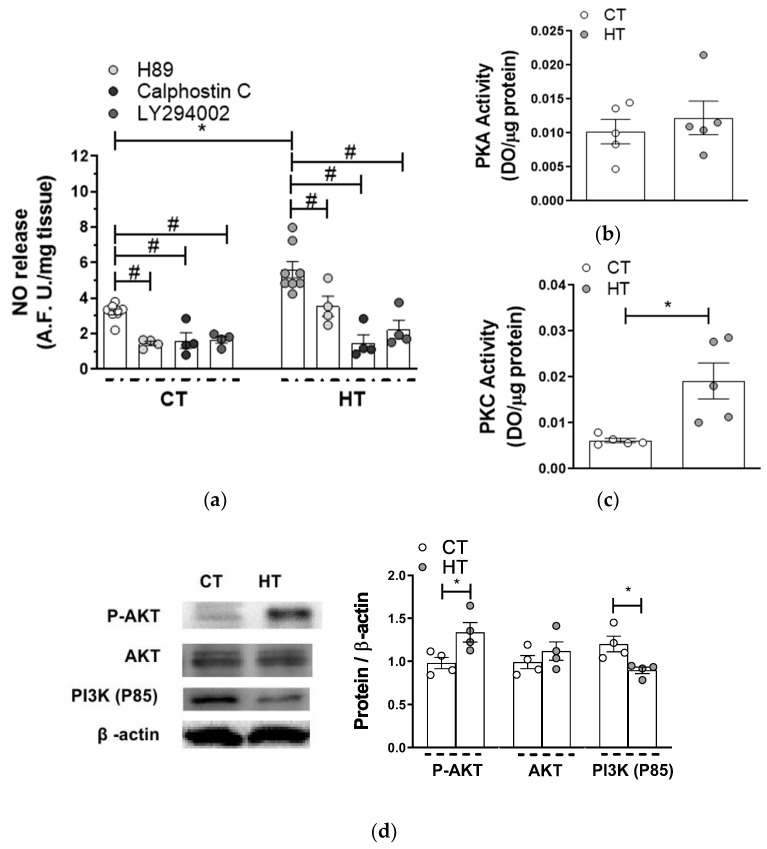
(**a**) Effect of preincubation with 1 µmol/L H89 (a PKA inhibitor), 0.1 µmol/L calphostin C (a PKC inhibitor) or 10 µmol/L LY 294002 (a PI3K inhibitor) on EFS-induced NO release in mesenteric arteries from control (CT) and hyperthyroidic rats (HT). Data (Mean ± S.E.M.) are expressed as arbitrary fluorescence units/mg tissue. * *p <* 0.05 CT vs. HT (Student´s *t*-test). # *p <* 0.05 conditions without inhibitor vs. conditions with inhibitor in each group (Student´s *t*-test). *n* = 4–8 segments in each experimental group. (**b**) PKA activity, and (**c**) PKC activity in mesenteric arteries from CT and HT rats. Results are represented as optical density (OD) units/µg protein (mean ± S.E.M). *n* = 5 segments from different animals in each group. * *p <* 0.05 CT vs. HT (Student´s *t*-test). (**d**) Western blot analysis for total AKT (AKT), phosphorylated AKT at the T308 residue (P-AKT) and P85 subunit of PI3K in mesenteric arteries from CT and HT rats. Each lane is representative of 4 isolated arterial segments from different animals in each group. Right panel shows densitometry analyses of the protein expression. Results (mean ± S.E.M) are expressed as protein expression relative to β-actin expression. * *p <* 0.05 CT vs. HT (Student’s *t*-test).

We observed that the expression of nNOS was reduced in arteries from HT rats, whereas its phosphorylation in the Ser1417 residue was enhanced by this pathology (Figure 5d). These results suggest that neuronal NO synthesis might be greater in arteries from HT animals. The fact that EFS-induced NO release was higher in mesenteric segments from HT rats confirmed this hypothesis (Figure 6a).

Several signaling transduction pathways are implicated in the phosphorylation and subsequent activation of nNOS, among which PKA, PKC and PI3K/AKT pathways play a relevant role in vascular tissue [27,35,36]. Preincubation with PKA inhibitor H89 (1 µmol/L) diminished EFS-induced NO release similarly in segments from both experimental groups (Figure 6a, Table 3), agreeing with the fact that PKA activity was similar in segments from both experimental groups (Figure 6b). Regarding PKC, preincubation with 0.1 µmol/L Calphostin C (a PKC inhibitor) decreased NO release more in segments from HT animals (Figure 6a, Table 3). In addition, PKC activity was greater in arteries from HT animals (Figure 6c). We also found that 10 µmol/L LY294002, a PI3K inhibitor, diminished EFS-induced NO release to a greater extent in arterial segments from HT animals. (Figure 6a, Table 3). Moreover, the expression of the PI3K regulatory subunit p85 was diminished in HT. AKT expression was similar in segments from both experimental groups, while AKT phosphorylation in its Thr308 residue was greater in arteries from HT rats (Figure 6d). To sum up, a greater PKC activity and a PI3K/AKT pathway hyperactivation could be responsible for the higher nNOS phosphorylation, the greater NO release and, consequently, the enhanced nitrergic function observed in HT.

## 3. Discussion

Hyperthyroidism is a common thyroid problem with multiple effects on almost all organs and systems in the body. An excess in thyroid hormones has marked metabolic effects, such as enhancement of oxygen consumption and thermogenesis, as well as increased lipid mobilization, promoting weight loss [37]. In addition, cardiovascular effects are the most common and dangerous effects of this pathology, being the main reason for the morbidity of the patients [5]. Therefore, understanding the cardiovascular alterations produced by hyperthyroidism is of great interest. Hence, we used an experimental hyperthyroidism model, consisting in a constant 14-day perfusion of L-Thyroxine, to analyze the possible role of perivascular innervation in the diminished vascular resistance present in this pathology. Exposition to L-Thyroxine induced a diminished body weight gain, probably linked to the lower adipose tissue accumulation, together with the enhanced citrate synthase activity in the soleus muscle, representative of the greater basal metabolic rate in the pathology [37,38,39]. This enhanced metabolic rate could increase protein catabolism and diminish muscle mass, as previously reported in both clinical and experimental models [38,39,40,41], while other studies showed no differences in skeletal muscle mass in patients with hyperthyroidism [42]. The soleus weight was not altered in the present study, probably due to the experimental model used in the present study. We also found a greater systolic blood pressure, reaching levels related to those present in hypertension [43], that could also be a consequence of thyroid hormone exposure [6,13]. Altogether, these results indicate that the rats exposed to L-Thyroxine developed some of the distinctive symptoms of hyperthyroidism and allowed us to validate this model for this pathology.

Despite the enhanced blood pressure in hyperthyroidism, diminished vascular resistance has been reported in this pathology [7], which might compensate for the great cardiac output present in hyperthyroidism. The mesenteric vasculature plays a relevant role in generating and maintaining vascular resistance [44]. Among the mechanisms implicated in the maintenance and regulation of vascular resistance, perivascular innervation has an important function in regulating vascular tone [21,22]. There is a rich functional innervation in SMA. Hence, we aimed to analyze whether HT could modify the vasoconstrictor response induced by EFS, which induces neurotransmitter release from nerve endings in the rat mesenteric artery. We observed a frequency-dependent contraction in segments from both CT and HT rats, this contraction being lower in HT animals. This diminished nervous function could participate in the reduced vascular resistance observed in hyperthyroidism and could be attributable to changes in the contractile capacity of the artery. Although McAllister et al. [45] reported a diminished KCl-induced constriction in hyperthyroidism, we observed no alterations in this response in our experimental procedure in agreement with those reported in different vascular beds and experimental procedures [27,46,47]. Thereby, alterations in arterial contractile machinery could be ruled out as responsible for our results.

Another factor responsible for the different EFS-induced contractile response could be an alteration in neurotransmitter function and/or release from perivascular innervation. Preincubation with TTX abolished this EFS-induced vasoconstriction in SMA from both CT and HT animals, confirming a neural origin for the EFS-induced contraction. Thereby, the differences observed in the EFS-induced vasomotor responses were the result of alterations in neurotransmitter participation produced by HT.

The SMA has plenty and functional sympathetic and nitrergic innervations. The participation of these different nervous components can be modified in different pathophysiological situations, including the exposure to T3 [27]. Regarding sympathetic function, most of the typical hyperthyroidism symptoms (tachycardia, sweating, lower adiposity, etc.) are a consequence of an adrenergic hyperstimulation. However, both increased and diminished plasmatic, urine and brain catecholamine levels have been reported in patients and experimental models of hyperthyroidism [28,29,37]. Given the essential role of sympathetic varicosities for blood pressure regulation [48], our next objective was to determine whether HT alters the sympathetic function in the SMA. For this purpose, we first analyzed the possible role of the adrenergic component of sympathetic innervation, by incubating the mesenteric segments with the alpha-adrenoceptor antagonist phentolamine. We observed that this drug significantly reduced the vasoconstrictor response elicited by EFS in segments from both CT and HT animals. A deeper analysis showed that this decrease was lower in HT animals, suggesting a blunted adrenergic function due to HT.

Noradrenergic neurotransmission consists of a balance between NA synthesis, NA release and the postsynaptic effect of NA. Both increases and decreases in NA release have been reported in HT [28,31]. In our experimental conditions, we observed a decrease in EFS-induced NA release in this pathological condition. TyrH and DβH are key enzymes implicated in NA synthesis. TyrH expression and activity were either enhanced or unaltered in nervous tissue and increased in cardiac tissue in different models of hyperthyroidism [49,50,51]. To the best of our knowledge, there is no report regarding possible modifications on DβH expression and/or activity in this pathology. When analyzing the components of this metabolic pathway, we observed that HT diminished TyrH expression and activation as well as DβH levels. Thereby, we can relate the decreased NA release observed in mesenteric arteries from HT to a diminished expression and activation of these enzymes implicated in its synthesis.

Regarding the vasoconstrictor response to exogenous NA, previous reports showed no modifications, with hypo- and hyper-reactivity to NA due to HT, the difference depending on the presence of the endothelium or the experimental model used [45,52,53,54]. In the present study, we found a similar NA-induced vasoconstriction in endothelium-denuded segments from both experimental groups, as McAllister reported in rat aorta [45].

Altogether, the results described above showed a reduced noradrenergic neurotransmission due to a diminished NA synthesis and release in HT animals. However, sympathetic neurotransmission involves simultaneous NA and ATP release, both acting on VSMCs [55,56,57]. The importance of ATP as a functional sympathetic neurotransmitter in blood vessels depends on the species, the vascular bed, the type of blood vessel and the pathology analyzed [58]. An evidence of purinergic neurotransmission has been reported in hyperthyroidism, showing enhanced P2 receptor expression in leukocytes and higher circulating ATP levels. These results are probably a consequence of the enhanced basal metabolic rate characteristic of this pathology [29]. Hence, we aimed to identify a possible differential role for neurogenic ATP in our experimental conditions. We observed a substantial phentolamine-resistant contractile response only in CT segments, which was abolished after adding the P2-purinoceptor antagonist suramin. What is more, suramin alone also decreased EFS-induced vasoconstriction only in mesenteric segments from CT animals. These results rule out a possible role for ATP in the neurogenic response in HT, therefore contributing in the decreased EFS-induced contraction observed in arteries from HT rats. Altogether, these data showed a diminished perivascular sympathetic function in mesenteric arteries from HT, due to decreased NA release and diminished purinergic function.

The results described above could explain by themselves the blunted EFS-induced vasoconstriction observed in SMA from HT rats. However, other perivascular innervation components might have a relevant role in this diminished constrictor response. The nitrergic component in mesenteric innervation has a vasodilator role that helps decrease the vasomotor response to EFS and can be altered by different physiopathological situations in which the hormonal status is modified [23,24,25,26]. Among the mechanisms by which thyroid hormones diminish vascular resistance, an increased production of both circulating and vascular NO has been described [8,10,30]. In line with this, we previously described that an acute incubation with T3 induced non-genomic effects that enhanced NO release and, consequently, nitrergic function [27]. Thus, we analyzed the possible alterations in the nitrergic component in HT rats. After preincubation with unspecific NOS inhibitor L-NAME, we observed an enhanced vasoconstriction in segments from both CT and HT, this increase being greater in HT segments. Thus, increased nitrergic function also seems to have a relevant role for the diminished EFS-induced contraction observed in arteries from HT animals.

Modifications in NO release or NO-dependent vasodilation could have a relevant role in the greater nitrergic function observed in SMA from HT rats. When analyzing EFS-induced NO release, we observed that it was higher in HT segments. This increased NO release could be associated with alterations in nNOS expression and/or activity [19,20]. Despite this, we observed that nNOS expression was lower in HT segments, not correlating with the enhanced NO release, but similar to that previously observed in hyperthyroidic rat aorta [32]. We previously described in this artery that an acute exposure to T3 increased nNOS activity in a non-genomic manner, through phosphorylation on its Ser1417 residue, which is responsible for nNOS activation [27]. Agreeing with this report, the phosphorylation of nNOS was greater in the segments from HT rats. This greater nNOS activity would explain the increase in NO release observed and make us hypothesize that the blunted nNOS expression might act as a compensatory mechanism for the enhanced activity of this enzyme.

There are several signaling transduction pathways implicated in the activation of nNOS, among which PKA, PKC and PI3K/AKT pathways play a relevant role in vascular tissue [27,35,36]. The activity of these pathways has been reported to increase in hyperthyroidism [1]. Hence, we aimed to determine the possible role of these pathways in the increased NO release in HT animals. We could observe that H89, a PKA inhibitor, diminished NO release similarly in both CT and HT segments, which correlates with the similar PKA activity observed in arteries in both experimental groups. Regarding PKC, we found that calphostin C decreased the EFS-induced NO release more in HT than in CT arteries. This result agrees with the fact that PKC activity was greater in arteries from HT animals. Concerning the PI3K/AKT signaling pathway, we also observed that preincubation with LY294002 diminished EFS-induced NO release in segments from both experimental groups, but more in arteries from HT animals. In parallel, in segments from HT rats, we observed a lower expression of P85, the regulatory subunit of PI3K, suggesting a higher activity for this enzyme in HT, as we previously described in the SMA after an acute ex vivo incubation with T3 [27]. The greater activation of AKT (through the phosphorylation on its Th308 residue) in arteries from HT rats, despite the similar expression of total AKT, confirms this hypothesis. To sum up, a greater PKC activity and a PI3K/AKT hyperactivation could be responsible for the higher nNOS phosphorylation, the greater NO release and, consequently, the enhanced nitrergic function observed in HT.

Once NO is released, it diffuses through the synaptic cleft to the vascular smooth muscle, triggering vasodilation through different mechanisms. Either increases or no alterations in smooth muscle sensitivity produced by hyperthyroidism have been previously reported [47,53,59,60]. We previously reported no alterations in the vasodilator response to NO donor DEA-NO after an acute exposure to T3 [27]. Since we found a similar DEA-NO-induced vasodilation in both CT and HT segments, we can rule out possible alterations in the smooth muscle sensitivity to NO induced due to hyperthyroidism.

To sum up, our results show that hyperthyroidism decreases EFS-induced vasoconstriction through: (1) a diminished sympathetic response due to a lower expression of TH and DβH, leading to a blunted NA synthesis and release; (2) a decreased purinergic neurotransmission; and (3) an increase in NO release from nitrergic nerve endings, through augmented nNOS activation due to nNOS hyperactivation of the PI3K-AKT and PKC pathways. Altogether, these alterations show a diminished role for perivascular mesenteric innervation in hyperthyroidism.

## 4. Materials and Methods

### 4.1. Animals

Male Wistar rats (*n* = 20; initial weight: 323.1 ± 13.49 g, Harlan Ibérica SL, Barcelona, Spain) were housed in the Animal Facility of the Universidad Autónoma de Madrid (Registration number EX-021U) and held in groups of 2 in appropriate cages, in controlled environmental conditions (20–24 °C, 55% relative humidity, 12 h light–dark cycle). The animals had access to fresh water and specific rat chow ad libitum.

Animals were randomly divided into two groups: (1) control rats (CT, *n* = 10); and (2) rats with induced hyperthyroidism (HT; *n* = 10). HT rats were infused with L-Thyroxine (1.5 µg/100 g per day, diluted in 0.02 N NaOH dissolved in sterile saline buffer for 14 days; Sigma-Aldrich Co., Madrid, Spain) with subcutaneously implanted Alzet osmotic minipumps (Durect Corp., Cupertino, CA, USA) [61]. Pumps containing only vehicle were implanted in CT rats.

### 4.2. Blood Pressure Measurements

At the end of the treatment period, systolic blood pressure (BP) was measured in conscious rats by a tail-cuff method (Letica, Digital Pressure Meter, LE5000, Barcelona, Spain) [15,20].

### 4.3. Animal Euthanasia and Sample Collection

After an overnight fasting, rats were euthanized by exsanguination by puncture of the infrahepatic inferior cava vein, after anesthesia (100 mg/kg ketamine hydrochloride,12 mg/kg xylazine; i.m.). After being euthanized, the soleus muscle was removed, weighed and immediately stored at −80 °C until citrate synthase analysis was performed. Visceral and epididymal adipose tissue pads were carefully collected. These fat pads are anatomically well defined and whole masses can be easily dissected. Left tibia length was also measured, since it is used to compare parameters in animals where the main variable in the model is body weight.

The SMA was carefully dissected, cleaned of connective tissue and maintained in cold (4 °C) Krebs–Henseleit solution (KHS) (in mmol/L: 115 NaCl, 25 NaHCO_3_, 4.7 KCl, 1.2 MgSO_4_∙7H_2_O, 2.5 CaCl_2_, 1.2 KH_2_PO_4_, 11.1 glucose and 0.01 Na_2_EDTA) bubbled with a 95% O_2_–5% CO_2_ mixture. Some segments were endothelium-denuded, quickly frozen in liquid nitrogen and maintained at −70 °C.

### 4.4. Citrate Synthase Activity

Citrate synthase activity, a marker of muscle oxidative activity, was determined in the right soleus as previously reported [62]. The enzyme activity was measured in whole muscle homogenates, and the complex resulting from acetyl-CoA and oxaloacetate was determined at 412 nm and 25 °C, within an interval of 10 min. Citrate synthase activity was expressed as nmol/min per mg of protein.

### 4.5. Vascular Reactivity

For vascular reactivity experiments, two parallel stainless-steel pins were introduced through the lumen of endothelium-denuded mesenteric segments (2 mm) and connected to a force transducer (Grass FTO3C; Quincy, MA, USA) and to a model 7D Grass polygraph [63]. Segments were suspended in an organ bath containing 5 mL of KHS (37 °C, bubbled with 95% O_2_–5% CO_2_ mixture, pH 7.4). and subjected to a tension of 4.9 mN, which was periodically readjusted (stabilization period). The vessels were exposed to 75 mmol/L KCl to check their functional integrity, obtaining a similar constrictor response in segments from both experimental groups (CT: 12.3 ± 0.9 mN; HT: 13.2 ± 1.5 mN; *p* > 0.05). After a washout period, the absence of the vascular endothelium was tested by the inability of 10 µmol/L acetylcholine (ACh) to relax segments precontracted with noradrenaline (NA).

For EFS experiments, segments were performed using a stimulator (Grass, model S44, Quincy, MA, USA) modified to supply the appropriate current strength. Frequency response curves to electrical field stimulation (EFS) were performed. The parameters used for EFS were 200 mA, 0.3 ms, 1–16 Hz, for 30 s with an interval of 1 min between each stimulus, the time required to recover basal tone. To evaluate whether the EFS-induced contractile response had a neural origin, the blocker for nerve impulse propagation tetrodotoxin (TTX, 0.1 µmol/L) was added to the bath 30 min before performing EFS-induced contraction. A washout period of at least 1 h was necessary to avoid desensitization between consecutive curves.

To determine the participation of the sympathetic and the nitrergic components of the mesenteric innervation in the EFS-induced response in segments from the CT and HT animals, 1 μmol/L phentolamine (alpha-adrenoceptor antagonist), 0.1 mmol/L suramin (a non-specific P2 purinergic receptor antagonist), a combination of phentolamine plus suramin or 0.1 mmol/L Nω-nitro-L-arginine methyl ester (L-NAME, non-specific inhibitor of nitric oxide synthase (NOS)) was added to the bath 30 min before performing the frequency response curve. Additionally, the vasomotor responses to exogenous NA (1 nmol/L–10 μmol/L) or to the NO donor diethylamine NONOate (DEA-NO, 0.1 nmol/L–0.1 mmol/L) were determined.

### 4.6. Neurotransmitter Release

Endothelium-denuded segments of rat SMA from CT and HT animals were stabilized. This was followed by two 10-min washout periods. Then, the medium was collected to measure basal neurotransmitter release. Afterwards, the organ bath was refilled and cumulative EFS periods were applied (see Vascular Reactivity section for parameters). Later, the medium was collected to measure the EFS-induced release.

Samples for NA release were collected in KHS buffer and analyzed using Noradrenaline Research EIA (Labor Diagnostica Nord, Gmbh and Co., KG, Nordhon, Germany). The assay was performed following the manufacturer’s instructions. Results were expressed as ng NA/mL per mg tissue. [62].

Samples for NO release were collected in HEPES buffer (in mmol/L: NaCl 119; HEPES 20; CaCl_2_ 1.2; KCl 4.6; MgSO_4_ 1; KH_2_PO_4_ 0.4; NaHCO_3_ 5; glucose 5.5; Na_2_HPO_4_ 0.15; pH 7.4; 37 °C) after incubation with the fluorescent probe 4,5-diaminofluorescein (DAF-2, 2 µmol/L), as previously described [24,62]. The fluorescence of the medium was measured at room temperature using a spectrofluorometer (Jenway 6280 Fluorimeter, Staffordshire, UK) with the excitation wavelength set at 492 nm and emission wavelength at 515 nm. The EFS-induced NO release was calculated by subtracting basal NO release from that evoked by EFS. Blank samples were collected in the same way from segment-free medium to subtract background emission. The amount of NO released was expressed as arbitrary fluorescence units/mg tissue. Some segments were incubated with 1 µmol/L H89 (a PKA inhibitor), 0.1 µmol/L calphostin C (a PKC inhibitor) or 10 µmol/L LY 294002 (a PI3K inhibitor). The different inhibitors were added to the bath 30 minutes before measuring the basal sample. The effect of each drug in NO release experiments was determined by calculating the percentage of inhibition produced by each drug.

### 4.7. PKA and PKC Activity Assays

PKA and PKC activities were, respectively, determined using a PKA kinase activity assay kit or a PKC kinase activity assay kit (Abcam, Cambridge, UK). Briefly, endothelium-denuded frozen arteries from both CT and HT animals were homogenized in a lysis buffer containing 1 mmol/L sodium vanadate, 1% SDS and pH 7.4, 0.01 mol/L Tris-HCl and centrifuged at 12,000× *g* for 10 min at 4 °C. The supernatant was then collected and used for the assay [35,36]. Assays were performed following the manufacturers’ protocols. Protein content was measured using a DC protein assay kit (BioRad, Madrid, Spain). Results were expressed as optical density (OD) units/μg protein.

### 4.8. Western Blot Analysis

Western blot analysis was performed as previously described [24,62]. Frozen segments without the endothelium were homogenized, and 30 µg protein was loaded in each lane. Mouse monoclonal antibody against tyrosine hydroxylase (TyrH, 1:500), rabbit P-TyrH (phospho S40) polyclonal antibody (1:1000), rabbit polyclonal anti-dopamine β-hydroxylase (DβH, 1:500); mouse monoclonal antibody against nNOS (1:2000), rabbit polyclonal anti-nNOS (neuronal) (phospho S1417) antibody (1:2000), rabbit polyclonal anti-PI 3 Kinase p85 beta antibody (1:500), rabbit polyclonal anti-pan-AKT antibody (1:1000) and rabbit polyclonal anti-pan-AKT (phospho T308) antibody (1:500) were used. Appropriate secondary antibodies were used (1:2000). The development and quantification of the images were performed using Quantity One software (v. 4.6.6, Biorad, Madrid, Spain). The same membrane was used to correct protein expression in each sample, by means of a monoclonal anti-β-actin−peroxidase antibody (1:50,000).

### 4.9. Drugs and Antibodies Used

L-Thyroxine, L-Noradrenaline hydrochloride, Ach chloride, diethylamine NONOate diethylammonium salt, TTX, L-NAME hydrochloride, phentolamine, suramin sodium salt, DAF-2, calphostin C, H89 and LY294002 were used. All drugs were purchased from Sigma-Aldrich (Madrid, Spain) except for LY294002 and H89, which were obtained from Tocris (Bristol, UK). Stock solutions (10 mmol/L) of drugs were made in distilled water, except for NA, which was dissolved in a NaCl (0.9%)-ascorbic acid (0.01% w/v) solution, DAF, H89, Calphostin C and LY294002, which were dissolved in dimethyl sulfoxide, and T4, which was dissolved in 0.02 N NaOH. These solutions were kept at −20 °C and appropriate dilutions were made in KHS on the day of the experiment. Mesenteric rings were incubated with the different vehicles in order to check that they did not affect basal tone.

Mouse monoclonal antibody against tyrosine hydroxylase was purchased from Santa Cruz Biotechnology (Dallas, TX, USA); rabbit P-TyrH (phospho S40) polyclonal antibody was purchased from Abnova (Taipei, Taiwan); mouse monoclonal antibody against nNOS was purchased from BD Biosciences (Madrid, Spain); rabbit polyclonal anti-nNOS (neuronal) (phospho S1417) antibody, rabbit polyclonal anti-PI 3 Kinase p85 beta antibody, rabbit polyclonal anti-pan-AKT antibody and rabbit polyclonal anti-pan-AKT (phospho T308) antibody were purchased from Abcam (Cambridge, UK); and rabbit polyclonal anti-dopamine β-hydroxylase and monoclonal anti-β-actin−peroxidase antibody were purchased from Sigma-Aldrich (Madrid, Spain). Anti-mouse and anti-rabbit secondary antibodies were purchased from GE Healthcare Systems (Madrid, Spain).

### 4.10. Data Analysis

Graph representation and statistical analysis were performed using GraphPad Prism 8.0 software (San Diego, CA, USA). The adipose tissue pads and heart weight were normalized using tibia length. The responses induced by EFS or NA were expressed as a percentage of the initial contraction elicited by 75 mmol/L KCl for comparison between experimental groups. To determine differences in the effect of preincubation with the different drugs in EFS-induced contraction experiments, we analyzed the differences between areas under the curve (dAUC). The relaxation induced by DEA-NO was expressed as a percentage of the initial contraction elicited by NA. The effect of each inhibitor in NO release experiments was determined by calculating the percentage of inhibition produced by each drug. Results were expressed as mean ± S.E.M. The EFS, NA or DEA-NO vasomotor responses were compared by means of an unpaired two-way analysis of variance (ANOVA) followed by a Bonferroni post hoc test. When comparing the effect of the different inhibitors on the EFS-induced contraction, we used a paired two-way ANOVA, followed by a Bonferroni post hoc test. For KCl, dAUC, NA, NO and PKA activity, PKC activity and Western blot analyses, the ROUT method was used to identify and remove outliers. Moreover, we applied a Shapiro–Wilk test to check the normality of the population data and, afterwards, we used a Student *t*-test. *p* < 0.05 was considered significant.

## 5. Conclusions

Hyperthyroidism is a chronic pathology, which causes a hyperdynamic cardiac state, leading to enhanced systolic blood pressure through sinus tachycardia, together with augmented inotropic and lusitropic effects. Conversely, hyperthyroidism is associated to reduced systemic vascular resistance as an attempt to compensate for the great cardiac output in the pathology. In the mesenteric vascular bed, perivascular innervation plays a major role in regulating vascular resistance. Our study provides, for the first time, an integrated analysis of the functional alterations in the different components of perivascular mesenteric innervation in hyperthyroidism. Our results show that hyperthyroidism decreases EFS-induced vasoconstriction through both a diminished sympathetic response and enhanced nitrergic participation. These results show that perivascular mesenteric innervation might play a relevant role in reducing the vascular resistance observed in hyperthyroidism.

## Figures and Tables

**Table 1:** Animal evolution.

	CT	HT
Body weight gain (g)	34.1 ± 4.6	21.3 ± 4.9 *
Tibia length (cm)	3.6 ± 0.2	3.7 ± 0.2
Systolic blood pressure (mm Hg)	107.1 ± 1.3	133.0 ± 3.0 *
Heart weight/tibia length (g/cm tibia)	0.31 ± 0.01	0.33 ± 0.01
Adipose tissue (g/cm tibia)	3.19 ± 0.1	2.43 ± 0.2 *
Soleus (g/cm tibia)	41.06 ± 3.2	41.72 ± 4.2
Citrate synthase activity (nmol/min mg protein)	31.9 ± 6.4	126.6 ± 49.7 *

All data are expressed as mean ± S.E.M. *n* = 5–10 rats in each group. * *p* < 0.05 control (CT) vs. hyperthyroidic (HT) rats (Student’s *t* test).

**Table 2:** EFS-induced contraction after preincubation with 0.1 µmol/L TTX in mesenteric segments from CT and HT rats.

	1 Hz	2 Hz	4 Hz	8 Hz	16 Hz
CT	2.1 ± 0.5	12.3 ± 3.4	28.8 ± 5.6	50.9 ± 5.5	66.9 ± 5.1
+ TTX	0	0	0	0.31 ± 0.1 *	2.5 ± 0.6 *
HT	0.66 ± 0.26	5.21 ± 1.19	15.45 ± 2.66	31.44 ±3.79	46.9 ± 4.7
+ TTX	0	0	0	0.2 ± 0.1 *	2.0 ± 0.2 *

Results (means ± SEM) are expressed as percentages of the response elicited by 75 mM KCl. Zeros are used when contraction was not detected. * *p* <0.05 vs. conditions without TTX (Two.way ANOVA followed by a Bonferrono post-hoc test). *n* = 10 animals in each group.

**Table 3:** Inhibition of EFS-induced NO release after preincubation with the PKA inhibitor H89 (1 µmol/L), with the PKC inhibitor Calphostin C (0.1 µmol/L) or with the PI3K inhibitor LY294002 (10 µmol/L).

	CT	HT
H89	58.6 ± 3.5	49.3 ± 5.4
Calphostin C	51.1 ± 10.9	80.8 ± 3.2 *
LY294002	52.7 ± 5.1	69.6 ± 4.2 *

Results (means ± SEM) are expressed as a percentage of inhibition produced by each drug. *n* = 4 segments from different animals. * *p <* 0.05 CT vs. HT (Student’s *t*-test).

## Data Availability

The data presented in this study are available on request from the corresponding author.

## Abbreviations

CT	Control rats
DβH	Dopamine β Hydroxylase
DAF	Diaminofluorescein
dAUC	Differences in area under the curve
DEA-NO	Diethylamine NONOate
EFS	Electrical field stimulation
HT	Hyperthyroidic rats
KHS	Krebs–Henseleit solution
L-NAME	Nω-Nitro-L-arginine methyl ester
NA	Noradrenaline
nNOS	Neuronal nitric oxide synthase
NO	Nitric oxide
PI3K	Phosphatydil inositol 3 phosphate kinase
PKA	Protein Kinase A
PKC.	Protein Kinase C
S.E.M.	Standard error of the media
SMA	Superior mesenteric artery
TyrH	Tyroxine hydroxylase
TTX	Tetrodotoxin
VSMC	Vascular smooth muscle cells
